# Immune cells within tertiary lymphoid structures are associated with progression‐free survival in patients with locoregional recurrent breast cancer

**DOI:** 10.1002/cam4.6864

**Published:** 2023-12-22

**Authors:** Jinyuan Gu, Jiaming Wang, Yue Sun, Xinrui Mao, Chao Qian, Xinyu Tang, Ji Wang, Hui Xie, Lijun Ling, Yi Zhao, Xiaoan Liu, Kai Zhang, Hong Pan, Shui Wang, Cong Wang, Wenbin Zhou

**Affiliations:** ^1^ Department of Breast Surgery The First Affiliated Hospital with Nanjing Medical University Nanjing China; ^2^ Jiangsu Key Lab of Cancer Biomarkers, Prevention and Treatment Jiangsu Collaborative Innovation Center For Cancer Personalized Medicine School of Public Health Nanjing Medical University Nanjing China; ^3^ Department of Oncology The First Affiliated Hospital with Nanjing Medical University Nanjing China; ^4^ Department of General Surgery Sir Run Run Hospital Nanjing Medical University Nanjing China; ^5^ Pancreas Center & Department of General Surgery The First Affiliated Hospital with Nanjing Medical University Nanjing Jiangsu China; ^6^ Pancreas Institute of Nanjing Medical University Nanjing Jiangsu China; ^7^ Department of Pathology The First Affiliated Hospital with Nanjing Medical University Nanjing China

**Keywords:** immune cells, locoregional recurrent breast cancer, prognostic predictor, progression‐free survival, tertiary lymphoid structures

## Abstract

**Introduction:**

Locoregional recurrent breast cancers have a poor prognosis. Little is known about the prognostic impact of immune microenvironment, and tertiary lymphoid structures (TLSs) in particular have not been reported. Thus, we aimed to characterize the immune microenvironment in locoregional recurrent breast tumors and to investigate its relationship with prognosis.

**Methods:**

We retrospectively included 112 patients with locoregional recurrent breast cancer, and hematoxylin–eosin staining and immunohistochemical staining (CD3, CD4, CD8, CD19, CD38, and CD68) were performed on locoregional recurrent tumor samples. The association of immune cells and TLSs with progression‐free survival (PFS) were analyzed by survival analysis.

**Results:**

We found more immune cells in the peritumor than stroma. After grouping according to estrogen receptor (ER) status, a low level of peritumoral CD3+ cells in ER+ subgroup (*p* = 0.015) and a low level of stromal CD68+ cells in ER− subgroup (*p* = 0.047) were both associated with longer PFS. TLSs were present in 68% of recurrent tumors, and CD68+ cells within TLSs were significantly associated with PFS as an independent prognostic factor (*p* = 0.035). TLSs and immune cells (CD3, CD38, and CD68) within TLSs were associated with longer PFS in ER− recurrent tumors (*p* = 0.044, *p* = 0.012, *p* = 0.050, *p* < 0.001, respectively), whereas CD38+ cells within TLSs were associated with shorter PFS in ER+ recurrent tumors (*p* = 0.037).

**Conclusion:**

Our study proposes potential predictors for the clinical prognosis of patients with locoregional recurrent breast cancer, emphasizing the prognostic value of immune cells within TLSs, especially CD68+ cells.

## 1 INTRODUCTION

Breast cancer is the most common malignant disease worldwide. A proportion of patients with primary breast cancer will still have a recurrence, and 8%–10% of these recurrent diseases are locoregional recurrences.^1^ Nielsen et al. have shown that the 5‐year probability of distant metastases is 73% after locoregional recurrence of breast cancer.^2^ The 5‐year overall survival rate after locoregional recurrence is 24.1%–59.9%.^3^ Patients with lymph node involvement, short recurrence interval, regional recurrence compared to local recurrence, discordant hormonal status with primary tumor or triple‐negative recurrent breast cancer, have a poor prognosis.^4^, ^5^, ^6^, ^7^ But not all patients with locoregional recurrences develop distant metastases, while some patients have a fairly good prognosis.^3^, ^8^ Therefore, predicting patients' prognosis with these factors is not sufficient and more indicators are needed.

Tumor microenvironment contains cancer cells, a repertoire of immune cells, stromal cells, endothelial cells, and cancer‐associated fibroblasts.^9^ The immune cells play important roles in tumorigenesis and progression and can be classified according to function into tumor‐antagonizing immune cells (such as effector T cells and M1‐polarized macrophages) and tumor‐promoting immune cells (such as regulatory T cells and myeloid‐derived suppressor cells),^10^ and also be classified according to histopathology into intratumoral (within the nest of malignant cells, with direct proximity to cancer cells), stromal (in the connective tissue and blood vessels surrounding the cancer nest), and peritumoral (around the tumor and can refer to cells at the advancing margin of the tumor, in the stroma or the tissues adjacent to the tumor) immune cells.^11^ Recently, the administrations of immune checkpoint inhibitors and adaptive immune cells have exhibited antitumor effect in multiple types of cancer, further emphasizing the role and function of immune cells in those patients.^12^, ^13^ Previous studies have found that tumor‐infiltrating lymphocytes (TILs) are associated with improved survival and TILs are reduced in recurrent tumors compared to primary breast cancer.^14^, ^15^ However, the tumor microenvironment continually changes over the course of cancer progression, and the composition and spatial architecture of tumor microenvironment are varied in different metastasis sites.^16^, ^17^, ^18^ To our knowledge, the profiling of immune cells in locoregional recurrent breast cancer has not been clearly described. Moreover, the previous study has found that post‐recurrence survival was significantly longer in patients with high TILs density in the recurrent tumor,^15^ with a small sample size and multiple metastasis sites. The relationship between different immune cells at different regions and prognosis of patients with recurrent disease remains inconclusive.

Tertiary lymphoid structures (TLSs), as important immune components, are ectopic lymphoid organs that develop in nonlymphoid tissues at sites of chronic inflammation including tumors, and have been found in the stroma, invasive margin, and/or core of different tumor types.^19^, ^20^ Many cell types are present in TLSs, such as T cells, B cells, plasma cells, and macrophages.^19^ A favorable impact of TLSs on prognosis of patients has been observed in a variety of tumors, including invasive breast cancer.^19^, ^21^, ^22^ TLSs are associated with high‐grade, abundant TILs infiltration, PD‐L1 expression on immune cells, and high pathological complete response after neoadjuvant chemotherapy in patients with breast cancer.^23^, ^24^, ^25^ However, the role of TLSs and immune cells within them in recurrent breast cancer has not yet been studied.

To the best of our knowledge, the immune microenvironment of locoregional recurrent breast cancer has not been clearly determined. The present study aimed at clarifying the immune microenvironment of locoregional recurrent breast cancer, in particular the different immune cell subsets in the stromal and peritumoral areas, as well as the TLSs and the immune cells within them. Importantly, the impact of these structures and immune cells on prognosis were also assessed.

## 2 MATERIALS AND METHODS

### 2.1 Study population

This was a retrospective cohort study of patients diagnosed with locoregional recurrent breast cancer. This study was conducted with the approval of the institutional ethics committee of The First Affiliated Hospital with Nanjing Medical University (approval no. 2022‐SR‐524), and the study was in compliance with the Helsinki Declaration. All patients provided written informed consent at the time of admission for their clinical data to be reviewed by us without exposing personal information.

From September 2008 to May 2016, patients were enrolled in this study when they met the following criteria: (1) Patient data available in our in‐patient' database; (2) Patients diagnosed with locoregional recurrent breast cancer in our hospital pathologically; (3) Systemic assessment was performed at initial diagnosis; (4) Locoregional recurrences with/without distant metastases were allowed; (5) Not receiving systemic treatment for recurrent disease at diagnosis of locoregional disease. Patients with Stage IV disease at initial diagnosis or those who were diagnosed with another coincident malignancy were excluded. The histopathological and clinical characteristics were obtained from our database, including age, estrogen receptor (ER) status, progesterone receptor (PR) status, human epidermal growth factor receptor 2 (HER2), and Ki67.

### 2.2 Immunohistochemistry (IHC)

Of the enrolled patients, surgical or core biopsy locoregional samples of recurrent tumors were obtained, and primary tumor samples were also available from 20 cases for pathological examinations. Formalin‐fixed, paraffin‐embedded (FFPE) tumor tissue was serially sectioned and stained with hematoxylin–eosin (HE) and IHC. All sections were examined by two pathologists independently who were blinded to the clinical characteristics or outcome of the patient.

For immunohistochemical experiments, 4‐μm‐thick sections were processed from each sample. All stainings were performed on whole sections. Sections were processed on automated immunostainers (Roche Benchmark XT). Antibodies used were purchased from Gene Tech (Shanghai) Company Limited, including anti‐CD3 (clone type: poly), anti‐CD4 (clone type: EP204), anti‐CD8 (clone type: SP16), anti‐CD19 (clone type: SP110), anti‐CD38 (clone type: 38C03), anti‐CD68 (clone type: KP1).

The ER and PR statuses for each sample were determined by IHC analysis. Given the large time span of enrolled patients, ER and PR positivity was defined as at least 10% of cells staining positive for ER or PR, respectively.

Due to the lack of criteria for immunohistochemical assessment of different subgroups of immune cells in tumors, this study adopted and modified previous scoring system for tumor‐infiltrating lymphocytes.^26^, ^27^, ^28^ Because intratumoral immune cells are typically present in small numbers and only detected in few cases, and they usually parallel to stromal immune cells,^26^ we did not evaluate intratumoral immune cells. There is currently no evidence showing whether immune cells at the tumor margin functionally differ from those located in the inner stroma.^26^ Therefore, we evaluated immune cells in two regions (Figure 2A): stromal immune cells (areas of tumor stroma around the cancer nest that contain immune cell infiltration but are not in direct contact with tumor cells) and peritumoral immune cells (the infiltrative margins surrounding the tumor).

All sections were digitized and loaded into CaseViewer (https://www.3dhistech.com/solutions/caseviewer/) or iViewer (http://upload.upathology.cn/iViewerSetup‐7.2.7.6alpha.exe) software for evaluation. Full‐face tumor sections were evaluated, with no focus on hotspots. Area with necrosis, hemorrhage, or crush artifacts was excluded. Immune cell scores were assessed by the percentage of the tumor area (both stromal area and peritumoral area) occupied by positively staining cells, using a continuous scale as a semiquantitative parameter in 10% increments; if less than 10%, a criterion of 5% was used. The degree of immune cell infiltration was classified it into four grades: 0 (0%); 1 (<5%); 2 (5%–20%); and 3 (>20%). Representative images of score were shown in Figure 2C.

### 2.3 TLSs detection

TLSs were identified by both H&E and IHC sections and the presence of TLSs were also reviewed by two pathologists independently. The assessment of TLSs was based on morphology. Rounded aggregates of organized lymphocytes appearing in the stroma, invasive margin, or core of tumors^19^ were identified as TLSs. The presence of immune cells (CD3+, CD4+, CD8+, CD19+, CD38+, and CD68+ cells) within TLSs were identified by IHC sections.

### 2.4 Clinical end point

The primary clinical outcome of this study was PFS. PFS was defined as the time from recurrent breast cancer diagnosis to disease progression, or the date when the patient died from any cause (including recurrent breast cancer).

### 2.5 Statistical analysis

A Paired Wilcoxon Signed Rank Test was used to compare IHC scores between the different groups, including regions (peritumoral and stromal) and paired primary and recurrent tumors. Kaplan–Meier method was used to analyze the association between PFS and clinical characteristics (site of recurrence and molecular subtype), IHC scores of immune cells (in peritumoral area, stromal area, and TLSs), and log‐rank test was used to investigate the significance of differences between these different groups. The univariate and multivariate Cox proportional hazards model was used to evaluate hazard ratios (HR) for the explanatory variables with 95% confidence intervals (CI) while these variables were tested the proportional hazards assumption for a Cox regression model fit.^29^ The variables were included in the multivariate Cox analysis while the *p*‐value of these variables in the univariate Cox analysis was <0.05 (Table S1). Data analyses were performed using statistical program R 4.2.0 (http://www.R‐project.org/) and packages “survival” were used. The level of statistical significance was set at 0.05.

## 3 RESULTS

### 3.1 Patient characteristics of locoregional recurrent breast cancer

In all, 112 patients with locoregional recurrent breast cancer were enrolled in this study. Patient characteristics were described in Table 1. The median age of patients at the time of diagnosis of recurrent tumors was 50 years, ranging from 23 to 91 years. Of these 112 patients, 77 (69%) had only locoregional recurrence, of which 38 (34%) had local recurrence and 39 (35%) had regional recurrence, and 35 (31%) had both locoregional and distant recurrences. 34 (31%) patients were ER+/HER2−, 27 (24%) were HER2+, and 25 (22%) were ER−/HER2−, while 26 (23%) patients could not be grouped into the above three types due to the lack of information. Ki‐67 was ≤20% in 21 (19%) patients and >20% in 64 (57%) patients. For treatment, 42 (37%) patients received radiotherapy after recurrence, 83 (74%) patients received chemotherapy, and 92 (82%) patients received surgery.

**TABLE 1:** Clinical characteristics of patients with recurrent breast cancer.

Parameter	*N* (%)
Total cohort	112 (100)
Age of recurrence, years	
Median	50
Range	23–91
Site of recurrence	
Locoregional recurrence	77 (69)
Local recurrence	38 (34)
Regional recurrence	39 (35)
Combined distant recurrence	35 (31)
ER status	
Positive	49 (44)
Negative	49 (44)
Unknown	14 (12)
PR status	
Positive	26 (23)
Negative	69 (62)
Unknown	17 (15)
HER2	
Positive	27 (24)
Negative	60 (54)
Unknown	25 (22)
Molecular subtype	
ER+/HER2−	34 (31)
HER2+	27 (24)
ER−/HER2−	25 (22)
Unknown	26 (23)
Ki‐67	
≤20%	21 (19)
>20%	64 (57)
Unknown	27 (24)
Radiotherapy for recurrence	
Yes	42 (37)
No	59 (53)
Unknown	11 (10)
Chemotherapy for recurrence	
Yes	83 (74)
No	23 (21)
Unknown	6 (5)
Surgery for recurrence	
Yes	92 (82)
No	20 (18)

### 3.2 Effect of clinical characteristics on the progression of recurrent breast cancer

From the time of confirmed recurrence, 6 (5%) patients were lost during follow‐up and 1 (1%) patient abandoned treatment. Of the remaining 105 patients, the median follow‐up time was 27 (range: 1–106) months. During follow‐up, 48 (46%) patients had disease progression and 4 (4%) patients died after progression.

**FIGURE 1:**
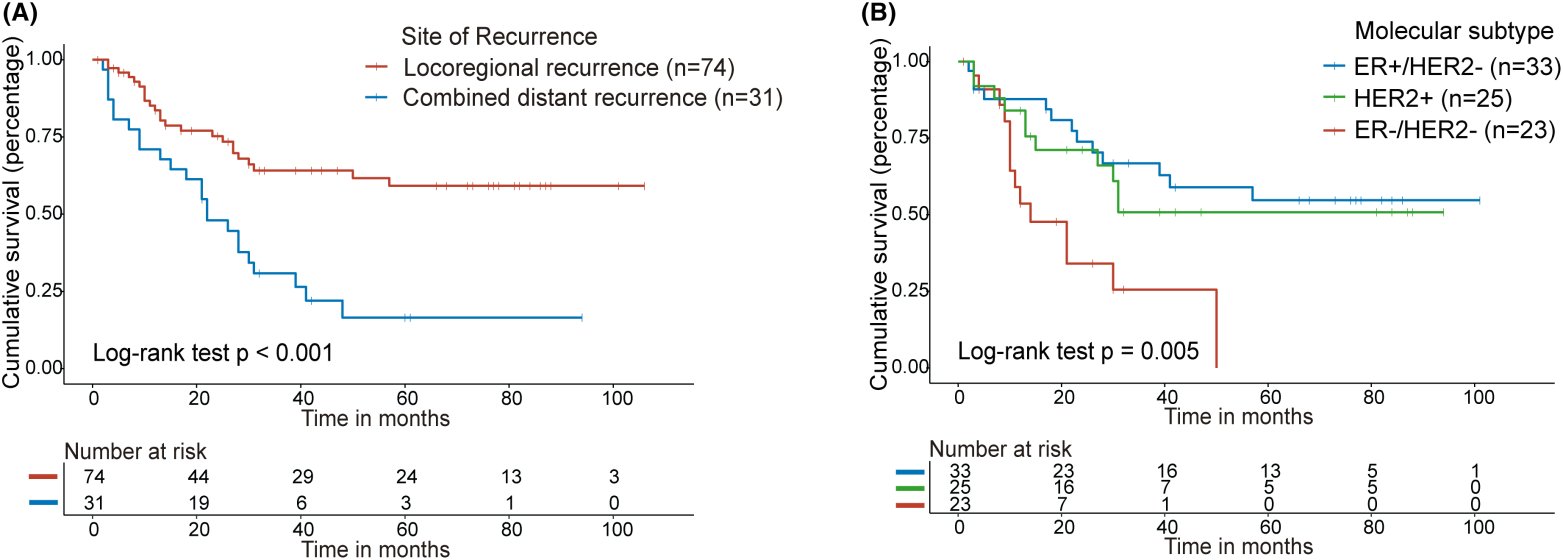
Kaplan–Meier analysis of progression‐free survival (PFS) in patients with locoregional recurrent breast cancer by site of recurrence and molecular subtype. (A) PFS survival curves stratified for site of recurrence. (B) PFS survival curves stratified for molecular subtype.

We first analyzed the relation between PFS and clinical characteristics. Patients with combined distant recurrence had a significantly shorter PFS (*p* < 0.001, Figure 1A) than those with only locoregional recurrence, and patients with ER−/HER2− disease also had a shorter PFS than other molecular subtypes (*p* = 0.005, Figure 1B). No significant association was identified between PFS and age of recurrence (*p* = 0.275, Table S1) or Ki‐67 (*p* = 0.424, Table S1).

Site of recurrence (HR: 0.359; 95% CI: 0.184–0.700; *p* = 0.003) and molecular subtype (ER+/HER2− vs. ER−/HER2−, HR: 0.334, 95% CI: 0.151–0.736, *p* = 0.007; HER2+ vs. ER−/HER2−, HR: 0.331, 95% CI: 0.147–0.745, *p* = 0.008) were independent prognostic factors (Table S2) by using multivariable Cox regression analysis.

### 3.3 Profile of immune cell infiltration in locoregional recurrent tumors

**FIGURE 2:**
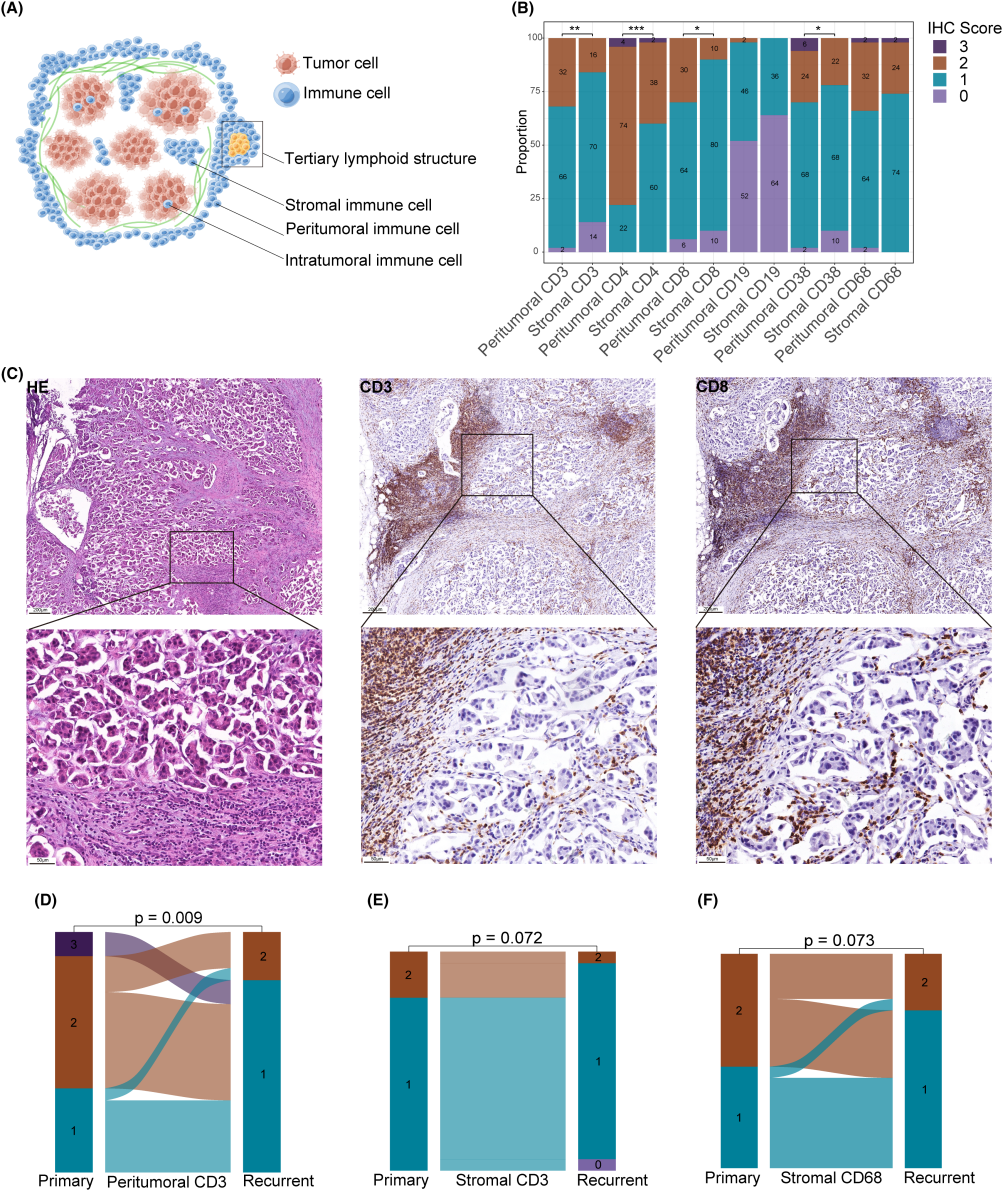
Distribution features of peritumoral and stromal immune cells of locoregional recurrent tumors and paired comparison with the primary tumors. (A) The schematic representation showing the location of stromal immune cells and peritumoral immune cells (by Figdraw). (B) Stacked barplots showing the distribution of subpopulations of immune cells in the peritumoral and stromal regions (*n* = 50). *p* values were calculated using Paired Wilcoxon Signed Rank Test (*p < 0.050; **p < 0.010; and ***p < 0.001). (C) Representative images of Immunohistochemistry (IHC) staining scores. Representative images of HE staining, CD3+ immune cell staining and CD8+ immune cell staining and magnified images of the boxed areas were shown, respectively. For CD3+ stained immune cells, peritumoral score 3 and stromal score 2. For CD8+ stained immune cells, peritumoral score 3 and stromal score 2. (D–F) Sankey plots showing the difference of IHC scores between paired primary and recurrent tumors (*n* = 20). Trends in peritumoral CD3+ cells (D), stromal CD3+ cells (E), and stromal CD68+ cells (F). *p* values were calculated using Paired Wilcoxon Signed Rank Test.

The immune cells were characterized based on their peritumoral and stromal regions within the tumor microenvironment to understand the role of the local immune system in locoregional recurrent breast cancer. Representative images of IHC staining scores of different immune cell subsets were shown in Figure 2A–C. CD3+ cells (*p* = 0.002), CD4+ cells (*p* < 0.001), CD8+ cells (*p* = 0. 011), and CD38+ cells (*p* = 0.042, Figure 2B) in the peritumoral area were significantly more than in the stroma, but other immune cell subsets showed a similar distribution trend without statistically significant differences.

Of these 112 patients, the specimens of the primary breast cancer were also obtained in 20 cases. Compared with the primary tumors, infiltration of stromal CD3+ cells and CD68+ cells was reduced in the recurrent tumors, but not statistically different (stromal CD3+ cells: *p* = 0.072; stromal CD68+ cells: *p* = 0.073, Figure 2E,F). Importantly, significantly reduced peritumoral CD3+ cells were observed in recurrent tumors than in primary tumors (*p* = 0.009, Figure 2D). Other immune cell subpopulations did not show significant differences in comparison with paired primary breast tumor (Figure S1).

### 3.4 Prognostic impact of immune cell infiltration in locoregional recurrent tumors

**FIGURE 3:**
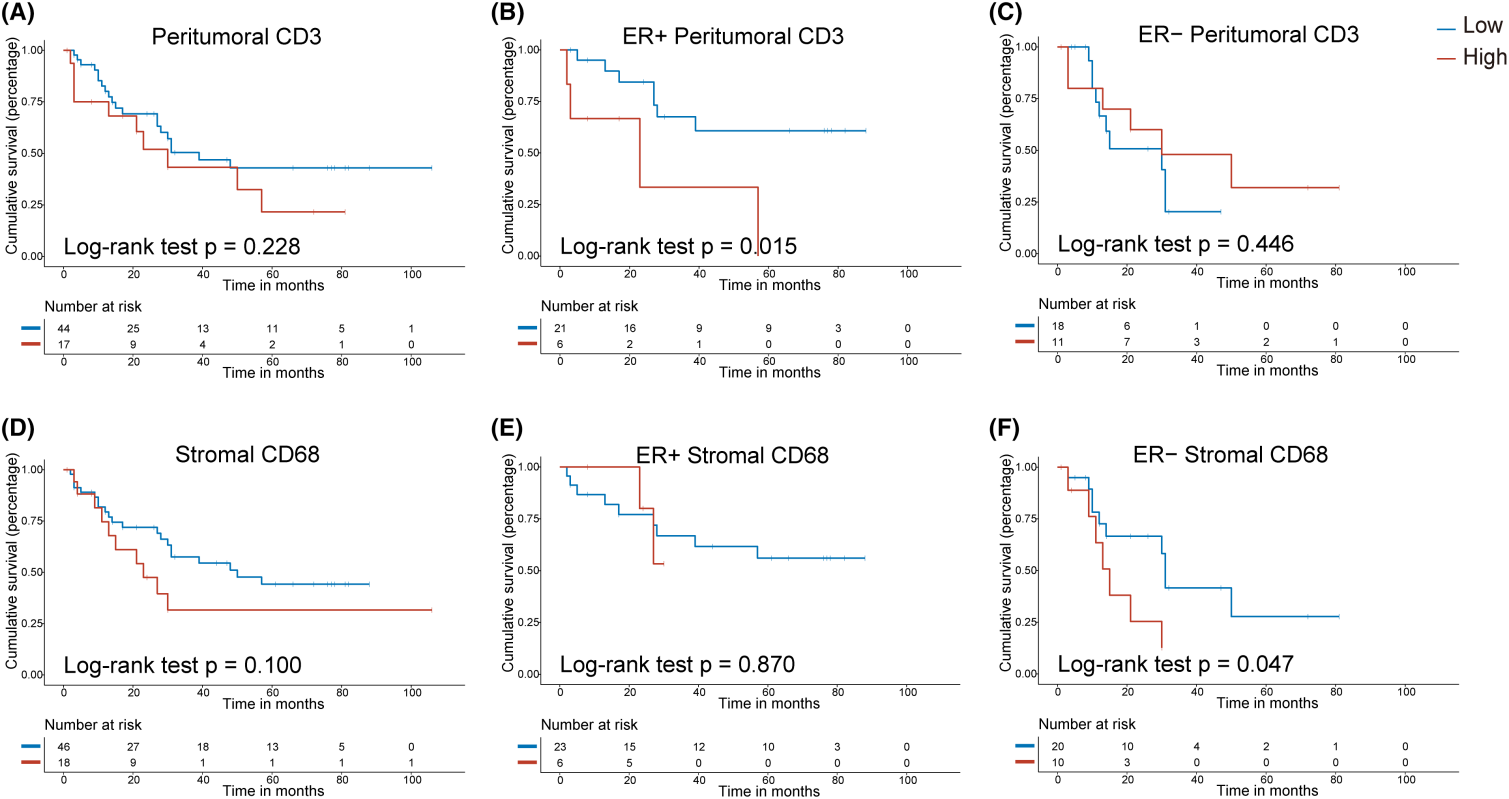
Immunohistochemistry (IHC) markers with prognostic features in the peritumoral and stromal regions. Depending on the proportion of immune cells IHC scores for different markers, we took the cutoff value closer to 50%. IHC scores 0–1 were classified as low infiltration and 2–3 as high infiltration. (A–C) Kaplan–Meier survival curves for progression‐free survival (PFS) by peritumoral CD3+ cells (A), and in ER+ subgroup (B) and ER− subgroup (C). (D–F) Kaplan–Meier survival curves for PFS by stromal CD68+ cells (D), and in ER+ subgroup (E) and ER− subgroup (F).

We next explored whether immune cell infiltration in the locoregional recurrent tumors would affect the prognosis. By univariate analysis, none of the individual immune cell markers was significantly associated with PFS (Figure 3A,D; Figure S2A–S2J). However, longer PFS was observed in patients with a low level of stromal CD68+ cells, in these 20 cases with paired specimens (*p =* 0.003, Figure S2K).

Because of the prominent association between ER status and immune infiltration phenotype, subgroup analyses were performed according to ER status (Figure 3). We found that a low level of peritumoral CD3+ cells was associated with longer PFS in the ER+ subgroup (*p =* 0.015, Figure 3B), but not associated with prognosis in the ER− subgroup (Figure 3C). Interestingly, a low level of stromal CD68+ was associated with longer PFS in the ER− subgroup (*p =* 0.047, Figure 3F) but not associated with prognosis in the ER+ subgroup (Figure 3E). No association was found between PFS and the other immune cell subsets regardless of the ER status (Figure S3). For the ER+/HER2− group, a low level of peritumoral CD3+ cells was associated with longer PFS (*p =* 0.034, Figure S4).

### 3.5 Tertiary lymphoid structures in locoregional recurrent tumors

**FIGURE 4:**
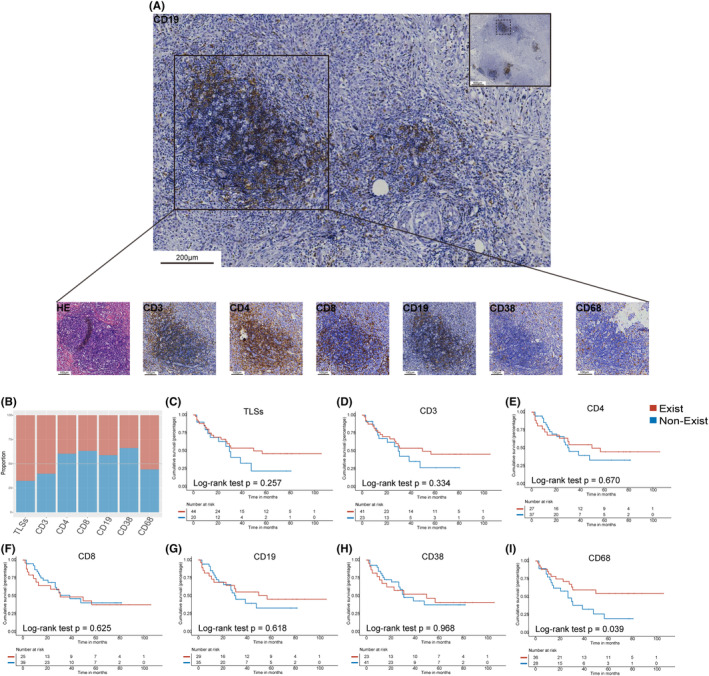
Existence of tertiary lymphoid structures (TLSs) and subpopulations of immune cells within TLSs in locoregional recurrent breast tumors. (A) Representative images of TLSs. The boxed area additionally showed H&E staining and singlet immunostaining of CD3, CD4, CD8, CD19, CD38, and CD68, respectively. (B) Stacked barplots showing the proportion of TLSs existing in locoregional recurrent tumors and the proportion of CD3+, CD4+, CD8+, CD19+, CD38+, and CD68+ cells existing within TLSs (*n* = 68). (C–I) Kaplan–Meier survival curves for progression‐free survival (*n* = 64) by existence or nonexistence of TLSs (C) and CD3+ cells (D), CD4+ cells (E), CD8+ cells (F), CD19+ cells (G), CD38+ cells (H), and CD68+ cells (I) within TLSs.

TLSs were detected in 68% of locoregional recurrent lesions (Figure 4A,B). Different immune cell subsets were present in different proportions in the focal TLSs. CD3+ cells were found in 60% of TLSs of locoregional recurrent tumors, and CD38+ cells were found in 34% of TLSs of locoregional recurrent tumors.

Importantly, patients with CD68+ cells in TLSs showed longer PFS (*p* = 0.039, Figure 4I) than those without them, while TLSs and other immune cells in TLSs had no prognostic impact (Figure 4C–H). To further validate the prognostic value of CD68+ cells in TLSs, we included site of recurrence and molecular subtype for multivariate Cox regression analysis as well. We found that the existence of CD68+ cells in TLSs was an independent prognostic factor in recurrent breast cancer, and PFS was significantly improved in patients with CD68+ cells in TLSs than in those without (HR: 0.411, 95% CI: 0.180–0.940, *p* = 0.035; Table S3).

### 3.6 Subgroup analysis of immune cells within TLSs in locoregional recurrent tumors

**FIGURE 5:**
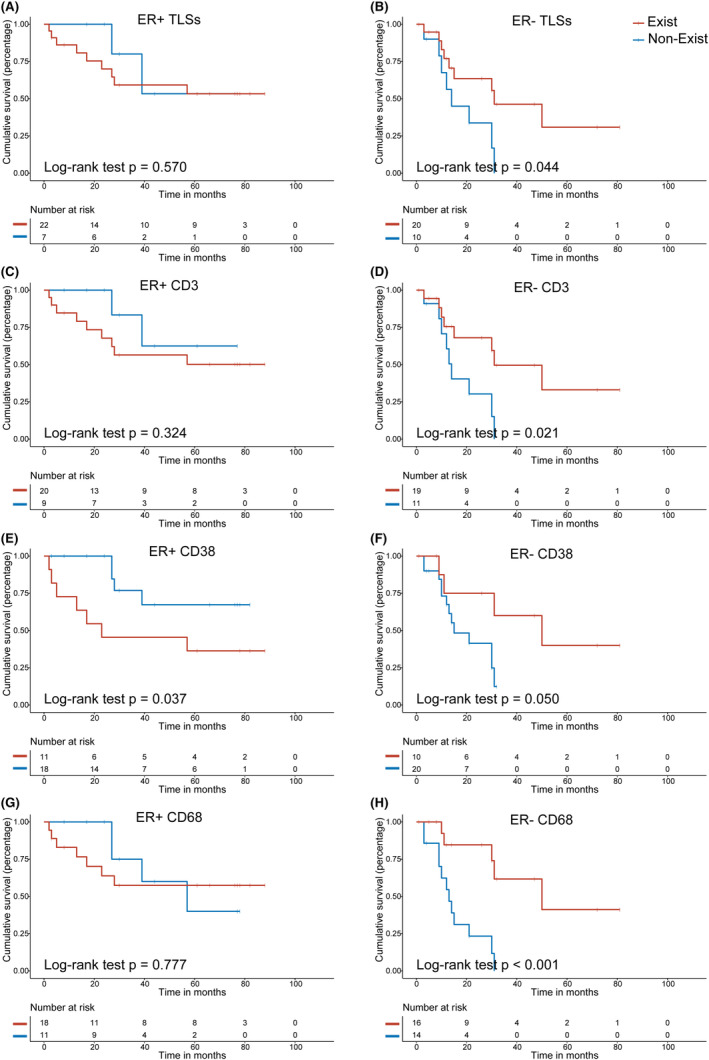
Relationship between tertiary lymphoid structures (TLSs) or immune cells within TLSs and progression‐free survival (PFS) in the ER+ and ER− subgroups. ER+ subgroups were shown on the left (A, C, E, G) and ER− subgroups were shown on the right (B, D, F, H). Kaplan–Meier survival curves for PFS in patients with TLSs (A, B), CD3+ cells in TLSs (C, D), CD38+ cells in TLSs (E, F), and CD68+ cells in TLSs (G, H).

In addition, we performed subgroup analyses of the relationship between immune cells within TLSs and prognosis (Figure 5). In the ER− subgroup, patients with TLSs (*p* = 0.044, Figure 5B), as well as with CD3+ cells (*p* = 0.021, Figure 5D) and CD68+ cells (*p* < 0.001, Figure 5H) in TLSs, had significantly longer PFS in recurrent tumors than those without them. However, in the ER+ subgroup, these structures and cell subsets had no prognostic value. Notably, CD38+ cells in TLSs were associated with longer PFS in ER− subgroup (*p* = 0.050, Figure 5F) but shorter PFS in ER+ subgroup (*p* = 0.037, Figure 5E). Other immune cell subsets in TLSs were not associated with PFS, regardless of ER status (Figure S5).

For HER+ and ER−/HER2− recurrent breast cancers, TLSs were associated with longer PFS (*p* = 0.047), with the similar results for CD3+ cells (*p* = 0.023), CD38+ cells (*p* = 0.041), and CD68+ cells (*p* < 0.001) in TLSs. In the ER+/HER2− subgroup, CD38+ cells in TLSs were associated with shorter PFS (*p* = 0.030, Figure S6).

## 4 DISCUSSION

To the best of our knowledge, this is the first study focused on the immune environment of locoregional recurrent breast cancer. More immune cells in the peritumor than in the stroma were observed, and TLSs were present in nearly two‐thirds of these patients. No significant difference was found between PFS and peritumoral or stromal immune cells in locoregional recurrent tumors. Importantly, CD68+ cells in TLSs served as an independent prognosticator of locoregional recurrent breast cancer. Moreover, in the ER− locoregional recurrent breast cancer, TLSs, and immune cells (CD3+, CD68+, and CD38+) within TLSs were associated with improved PFS.

Immune cells are essential components of tumor environment, reflecting the immunogenicity of tumor, and they are important biomarkers for chemotherapy and immunotherapy^30^, ^31^ for patients with incurable recurrent breast cancer. It has been reported that in invasive breast cancer there is no difference in the distribution of immune cells between the inner stroma and invasive edge of tumor,^32^ but in our study, peritumoral immune cells were more abundant than stromal in locoregional recurrent breast cancer. Specifically, we found that CD3+, CD4+, CD8+, and CD38+ cells were more in the peritumor than in the stroma. Previously studies have shown a decreasing trend of TILs in metastatic/recurrent breast cancer compared to primary breast cancer,^15^, ^26^, ^33^, ^34^ and we also found this decreasing trend both in the peritumor and stroma of locoregional recurrent tumors. Moreover, it has been suggested that less TILs in recurrent breast cancers are associated with a poor post‐recurrence survival.^15^ This study investigated the relationship between infiltration of different immune subpopulations at different areas in locoregional recurrent breast cancer and patient prognosis, and no significant associations were achieved. Future studies are still needed to determine the role of immune cells in recurrent tumors.

TLSs are organized aggregates of immune cells that form postnatally in nonlymphoid tissues.^35^ Many studies have shown that the presence of TLSs in tumors correlates with better survival and clinical outcome upon immunotherapy.^21^, ^22^, ^35^, ^36^, ^37^, ^38^ However, the presence and prognostic value of TLSs in locoregional recurrent breast cancer, to our knowledge, has not been studied. We found that TLSs were present in the majority of recurrent patients and that the distribution of immune cells within TLSs varied considerably. Among them, CD3+ cells were most present, while CD38+ cells were least present. Interestingly, by Kaplan–Meier method we also found that patients with CD68+ cells in TLSs had a longer PFS compared to those without. And we also did Cox analysis for this variable and included the site of recurrence and molecular subtype as adjustments in the multivariate Cox analysis. CD68+ cells in TLSs was a potential predictor for patients with locoregional recurrent breast cancer.

CD68 was a marker of tumor‐associated macrophages, and was associated with both favorable and unfavorable outcomes.^39^, ^40^, ^41^ And in HR+/HER2− breast tumors, higher level of CD68+ cells was associated with favorable response to neoadjuvant chemotherapy.^42^ We emphasized the importance of CD68+ cells in TLSs, which were present in about half of patients with locoregional recurrent breast cancer and were associated with improved PFS, especially in ER− patients. However, also in ER− patients, a low level of stromal CD68+ was associated with longer PFS. The reason for this might be the heterogeneity of CD68+ cells across different sites of the tumor microenvironment. CD68+ cells could be detected by IHC, flow cytometry, RNA‐Seq, and so on, but in specific sites, such as TLSs and peritumoral region, they could be evaluated by IHC. Tumor‐associated macrophages were roughly categorized into two different subsets named inflammatory M1 and anti‐inflammatory M2 macrophages.^43^ A variety of drugs such as histone deacetylase inhibitors and toll‐like receptors agonists have now been shown in preclinical models to enhance cancer therapy by increasing macrophage infiltration or the M1/M2 ratio.^44^, ^45^, ^46^

An earlier study by Denkert et al. has shown that increased TILs are associated with a survival benefit in HER2‐positive breast cancer or TNBC but are an adverse prognostic factor for survival in lunimal‐HER2‐negative breast cancer.^47^ Moreover, a study by Cesar Augusto Santa‐Maria et al. also has shown that TNBC respond better to immune checkpoint inhibitors compared with ER+ breast cancer.^48^ These suggest that breast cancers with different subtypes have different tumor immune microenvironments. Therefore, we assessed the prognostic relevance of immune cells within TLSs according to ER status. In the ER− subgroup, patients with TLSs, as well as with CD3+ cells and CD68+ cells in TLSs, had significantly longer PFS in recurrent tumors than those without. However, in the ER+ subgroup, TLSs and these cell subsets had no prognostic value. Interestingly, we found that the presence of CD38+ cells in TLSs had a worse prognosis in ER+ recurrent tumors than those without, but a better prognosis in the more aggressive ER−tumors. Similar results were obtained by subgroup analyses according to molecular subtypes. The same results were also found in the univariate Cox analyses (Table S4), but given that these results were already stratified according to the ER status and the small simple size after stratification, we did not perform further multivariate analyses. One possible explanation for this difference could be the contribution of different phenotypes of CD38+ cells in ER+ and ER− tumors. A population of CD38+ cells expressing IgA, IL‐10, and PD‐L1 have been identified in prostate cancer^49^ and hepatocellular carcinomas,^50^ and have been shown to suppress CD8+ T cells activation and exhibit immunosuppressive effects. Perhaps this group of CD38+ cells could be found more in ER+ breast tumors than in ER− tumors. The different prognostic role of CD38+ cells within TLSs in different ER status found in this study may provide an explanation for the differential efficacy of chemotherapy and immunotherapy for breast cancer. Therefore, future studies according to hormone receptor status are needed to better characterize the prognostic relevance of immune cells within TLSs.

Several limitations existed in the current study. First, our sample size was not large enough, resulting in a small number of variables with *p* < 0.050, which were also not suitable for correction, and a larger sample size was needed for subsequent validation of the results. Second, this was a single‐center study, which could lead to potential selection bias, but all of our enrolled patients were included according to the inclusion criteria, and thus we included a relatively homogeneous group of patients in the overall population of recurrent breast cancer patients. Third, the sample we included in the study spanned a long period of time, and a very small number of patients, especially with disease progression after first‐line treatment, were lost to follow‐up within a short period of time after treatment, and the overall survival was difficult to obtain. Fourth, this study was a retrospective cohort study, and the results were influenced by many factors that might have information bias.

In conclusion, this study comprehensively characterized immune microenvironment in locoregional recurrent breast cancer and for the first time showed the correlation between immune subpopulations in TLSs and prognosis, which might provide novel prognostic markers for recurrent tumors. We highlighted the importance of CD68+ cells within TLSs for PFS in locoregional recurrent breast cancer. Moreover, TLSs, CD3+ cells, and CD38+ cells in TLSs are associated with a good prognosis in patients with ER− recurrent breast cancer. Despite these observations, further studies are needed to confirm these findings.

## AUTHOR CONTRIBUTIONS

**Jinyuan Gu:** Data curation (equal); methodology (equal); resources (equal); writing – original draft (equal). **Jiaming Wang:** Data curation (equal); resources (equal); writing – original draft (equal). **Yue Sun:** Data curation (equal); investigation (equal); writing – original draft (equal). **Xinrui Mao:** Data curation (equal); resources (equal); validation (equal); writing – original draft (equal). **Chao Qian:** Data curation (equal); investigation (equal); resources (equal); validation (equal). **Xinyu Tang:** Formal analysis (equal); validation (equal); visualization (equal); writing – original draft (equal). **Ji Wang:** Resources (equal); software (equal); validation (equal); writing – original draft (equal). **Hui Xie:** Project administration (equal); writing – review and editing (equal). **Lijun Ling:** Project administration (equal); writing – review and editing (equal). **Yi Zhao:** Project administration (equal); writing – review and editing (equal). **Xiaoan Liu:** Project administration (equal); writing – review and editing (equal). **Kai Zhang:** Formal analysis (equal); methodology (equal); software (equal); validation (equal); visualization (equal). **Hong Pan:** Conceptualization (equal); project administration (equal); writing – review and editing (equal). **Shui Wang:** Conceptualization (equal); funding acquisition (equal); project administration (equal); writing – review and editing (equal). **Cong Wang:** Conceptualization (equal); methodology (equal); project administration (equal); visualization (equal); writing – review and editing (equal). **Wenbin Zhou:** Conceptualization (equal); funding acquisition (equal); project administration (equal); validation (equal); writing – review and editing (equal).

## FUNDING INFORMATION

This work was supported in part by the National Natural Science Foundation of China (81771953 and 82172683) and a project funded by the Priority Academic Program Development of Jiangsu higher Education Institutions.

## CONFLICT OF INTEREST STATEMENT

The authors declare no conflicts of interest.

## ETHICS STATEMENT

The study was conducted with the approval of the institutional ethics committee of The First Affiliated Hospital with Nanjing Medical University (approval no. 2022‐SR‐524), and the study was in compliance with the Helsinki Declaration. All patients provided written informed consent for their clinical data to be reviewed.

## Supporting information


Figure S1.
Figure S2.
Figure S3.
Figure S4.
Figure S5.
Figure S6.

Table S1.

Table S2.

Table S3.

Table S4.

## Data Availability

The data that support the findings of this study are available on request from the corresponding author.
