# Supplementary material for: Immune cells within tertiary lymphoid structures are associated with progression‐free survival in patients with locoregional recurrent breast cancer
Source: Cancer Med. 2023 Dec 22;13(1):e6864. doi: 10.1002/cam4.6864 (PMC10807640; doi:10.1002/cam4.6864)
Supplement: Supplementary file 1 — Figure S1. Figure S2. Figure S3. Figure S4. Figure S5. Figure S6. [file CAM4-13-e6864-s001.pdf]

Supplementary materials:

Immune cells within tertiary lymphoid structures are associated with progression-free survival in patients with locoregional recurrent breast cancer

Jinyuan Gu, Xinrui Mao, Yue Sun, Jiaming Wang, Chao Qian, Xinyu Tang, Ji Wang, Hui Xie, Lijun Ling, Yi Zhao, Xiaoan Liu, Kai Zhang, Hong Pan, Shui Wang, Cong Wang, Wenbin Zhou

Table of contents:

1. Supplementary Figure S1
2. Supplementary Figure S2
3. Supplementary Figure S3
4. Supplementary Figure S4
5. Supplementary Figure S5
6. Supplementary Figure S6
7. Supplementary Table S1
8. Supplementary Table S2
9. Supplementary Table S3
10. Supplementary Table S4

Supplementary Figure S1

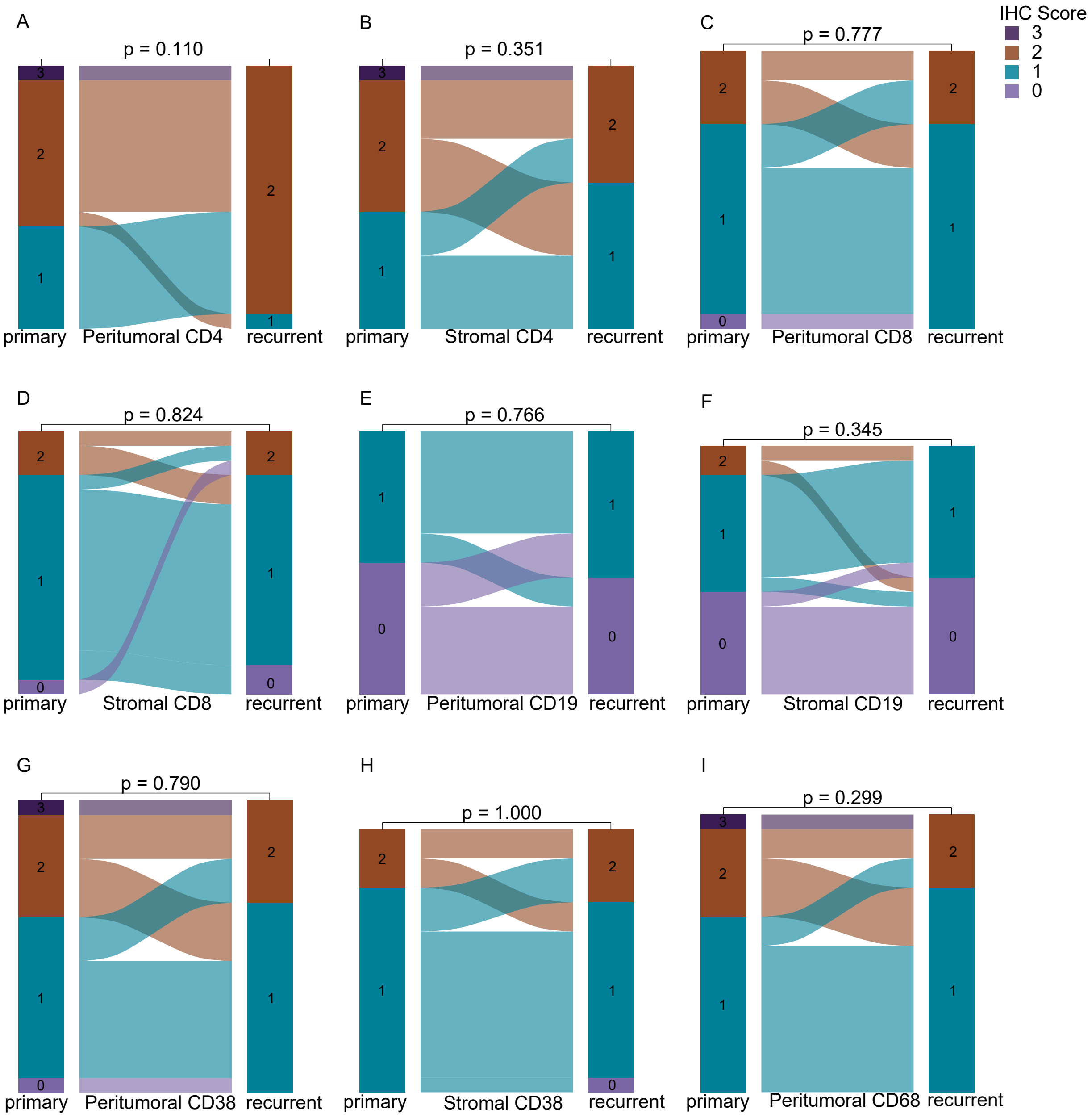

Supplementary Figure S1. Sankey plots showing the difference of IHC scores between paired primary and recurrent tumors (n=20). Including the trend of peritumoral CD4+ cells **(A)**, stromal CD4+ cells **(B)**, peritumoral CD8+ cells **(C)**, stromal CD8+ cells **(D)**, peritumoral CD19+ cells **(E)**, stromal CD19+ cells **(F)**, peritumoral CD38+ cells **(G)**, stromal CD38+ cells **(H)**, peritumoral CD68+ cells **(I)**. P values were calculated using Wilcoxon matched-pairs signed-ranks test.

Supplementary Figure S2

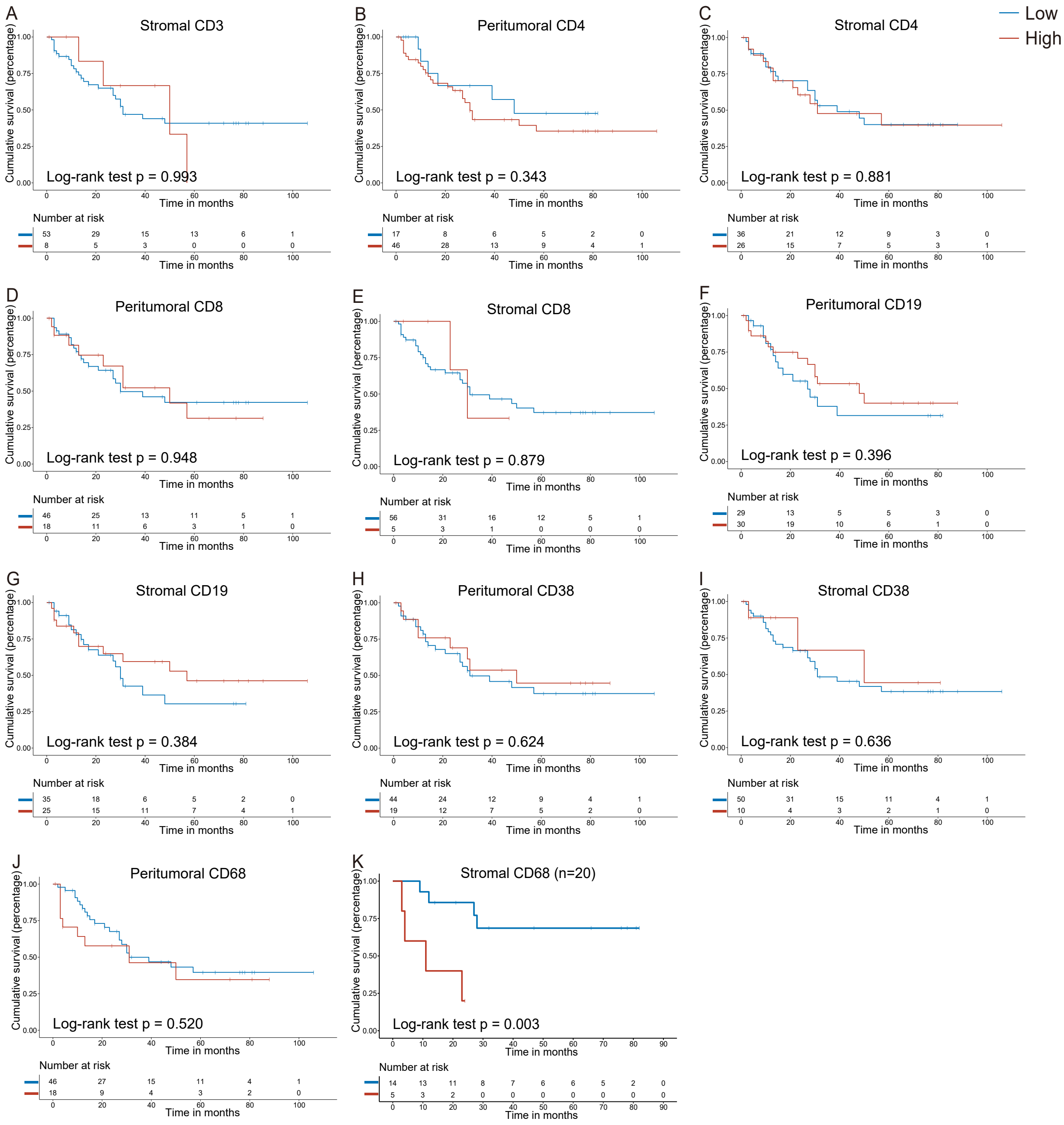

Supplementary Figure S2. Survival analysis of different positive immune cells.
IHC scores of CD19+ cells were classified as 0 for low infiltration and 1-2 for high infiltration. IHC scores of other positive immune cells were classified as 0-1 for low infiltration and 2-3 for high infiltration. Kaplan–Meier survival curves for PFS by stromal CD3+ cells **(A)**, peritumoral CD4+ cells **(B)**, stromal CD4+ cells **(C)**, peritumoral CD8+ cells **(D)**, stromal CD8+ cells **(E)**, peritumoral CD19+ cells **(F)**, stromal CD19+ cells **(G)**, peritumoral CD38+ cells **(H)**, stromal CD38+ cells **(I)**, and peritumoral CD68+ cells **(J)**. **(K)** Kaplan–Meier analysis of PFS in patients with locoregional recurrent breast cancer stratified for stromal CD68+ cells in 20 cases with paired specimens.

Supplementary Figure S3

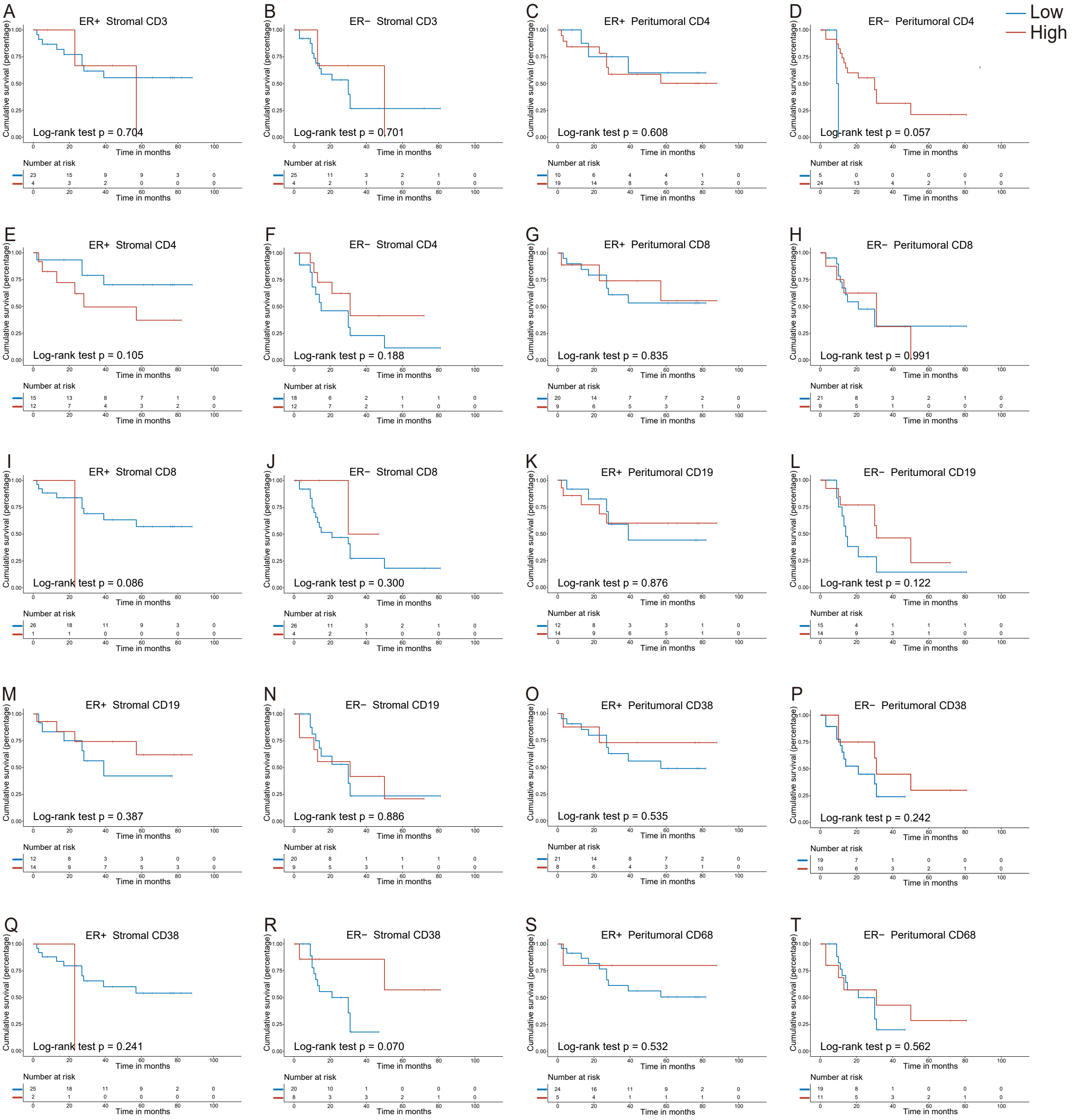

Supplementary Figure S3. Relationship between different positive immune cells and PFS in the ER+ and ER- subgroups. IHC scores of CD19+ cells were classified as 0 for low infiltration and 1-2 for high infiltration. IHC scores of other positive immune cells were classified as 0-1 for low infiltration and 2-3 for high infiltration. Kaplan–Meier survival curves for PFS by stromal CD3+ cells **(A-B)**, peritumoral CD4+ cells **(C-D)**, stromal CD4+ cells **(E-F)**, peritumoral CD8+ cells **(G-H)**, stromal CD8+ cells **(I-J)**, peritumoral CD19+ cells **(K-L)**, stromal CD19+ cells **(M-N)**, peritumoral CD38+ cells **(O-P)**, stromal CD38+ cells **(Q-R)**, and peritumoral CD68+ cells **(S-T)**.

Supplementary Figure S4

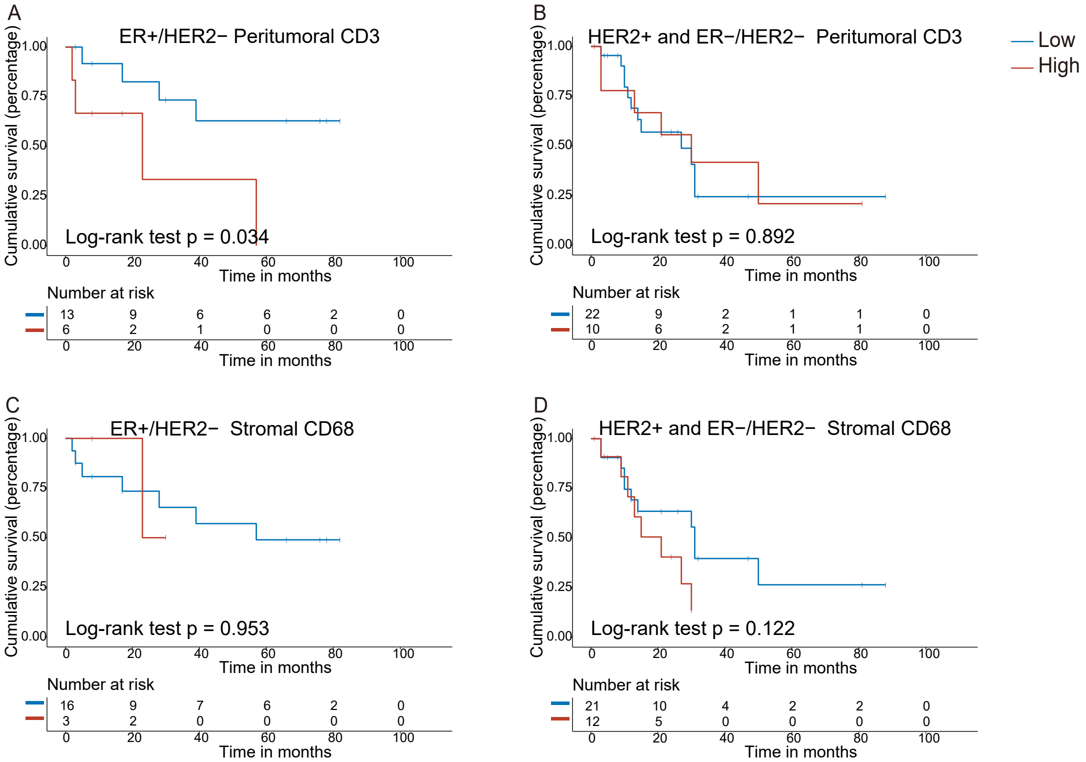

Supplementary Figure S4. Validation of IHC markers with prognostic features in the peritumor and stroma by different molecular subtype.

IHC scores 0-1 were classified as low infiltration and 2-3 as high infiltration. Kaplan–Meier survival curves for PFS by peritumoral CD3+ cells in ER+/HER2- subgroup **(A)**, HER2+ and ER-/HER2- subgroup **(B)**, and stromal CD68+ cells in ER+/HER2- subgroup **(C)**, HER2+ and ER-/HER2- subgroup **(D)**.

Supplementary Figure S5

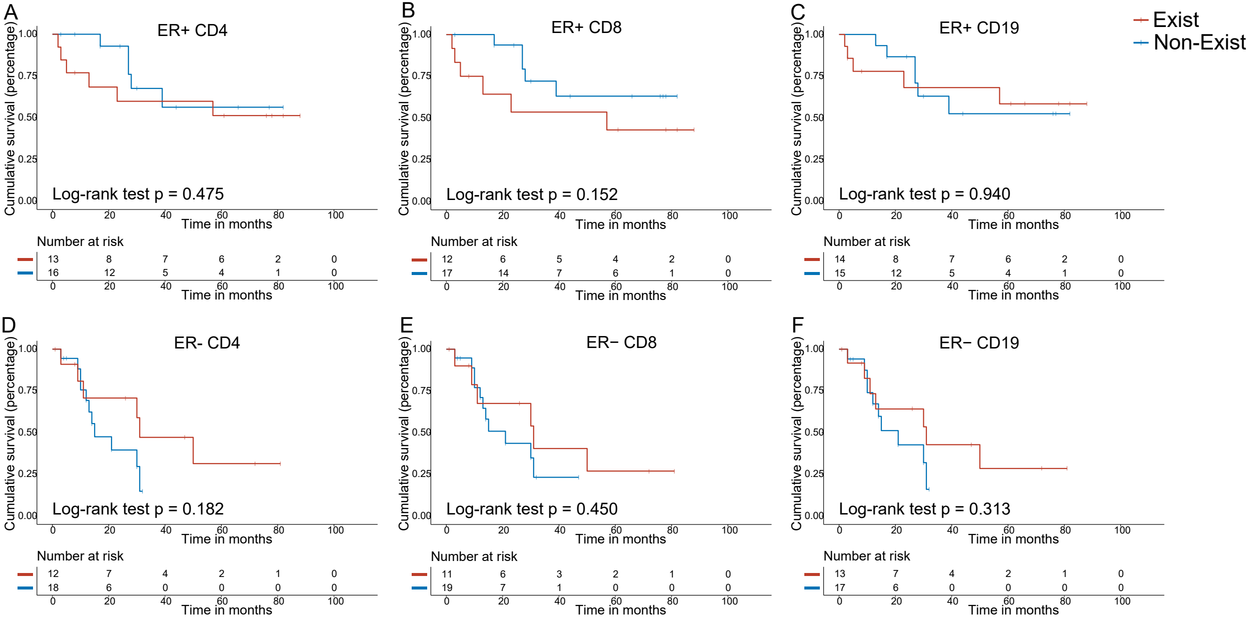

Supplementary Figure S5. Relationship between existence of immune cells in TLSs and PFS in the ER+ and ER- subgroups. ER+ subgroups were shown on the top **(A-C)** and ER- subgroups were shown on the bottom **(D-F)**. Kaplan-Meier survival curves for PFS in patients with CD4+ cells in TLSs **(A, D)**, CD8+ cells in TLSs **(B, E)** and CD19+ cells in TLSs **(C, F)**.

Supplementary Figure S6

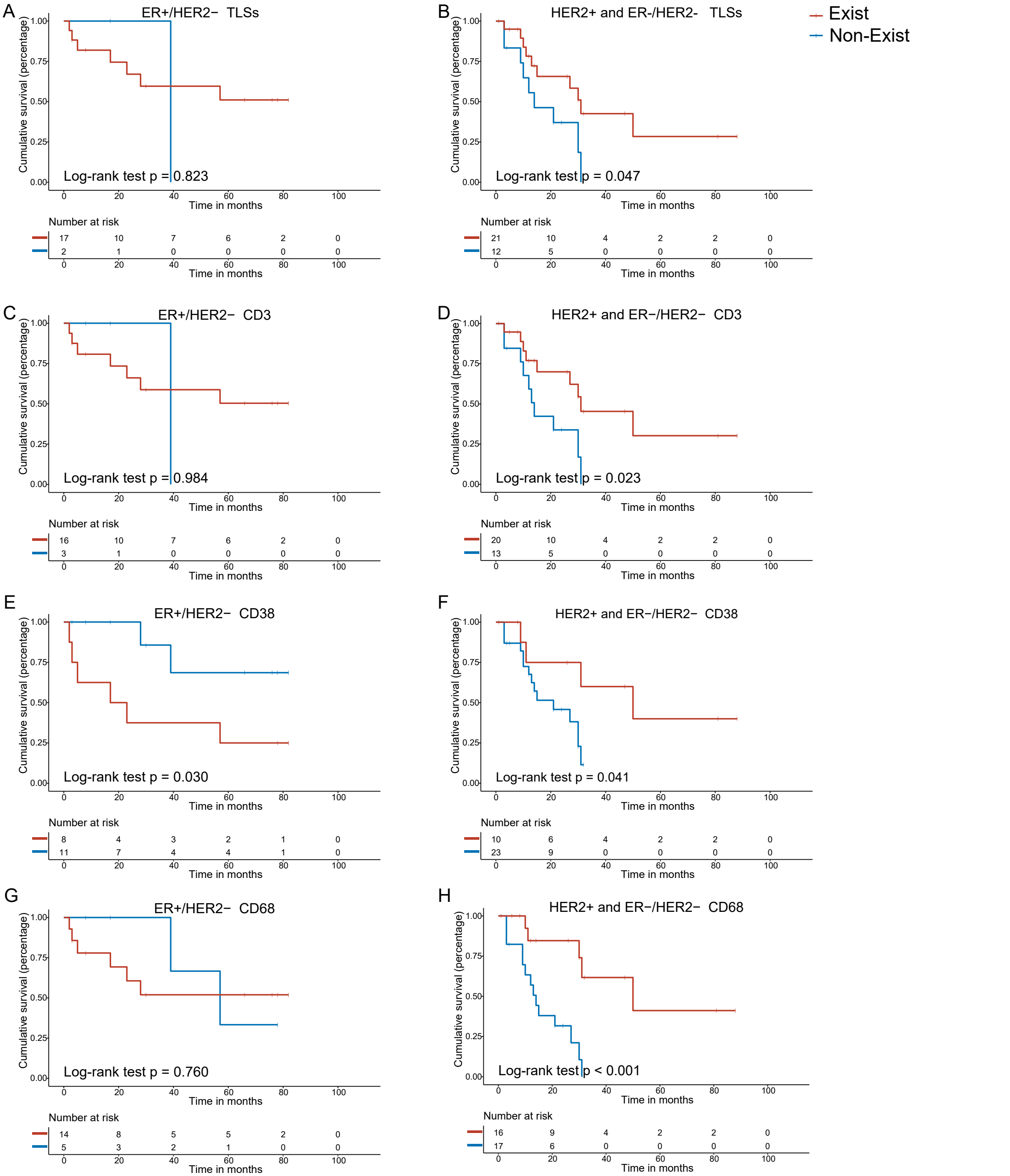

Supplementary Figure S6. Validation relationship between TLSs or immune cells within TLSs and PFS by different molecular subtype. ER+/HER2- subgroups were shown on the left **(A, C, E, G)** and HER2+ and ER-/HER2- subgroups were shown on the right **(B, D, F, H)**. Kaplan-Meier survival curves for PFS in patients with TLSs **(A-B)**, CD3+ cells in TLSs **(C-D)**, CD38+ cells in TLSs **(E-F)** and CD68+ cells in TLSs **(G-H)**.
